# Clinical impact of cardiovascular disease on patients with bronchiectasis

**DOI:** 10.1186/s12890-020-1137-7

**Published:** 2020-04-23

**Authors:** Shanshan Chen, Aimin Qiu, Zhang Tao, Hailin Zhang

**Affiliations:** Department of Respiratory and Critical Care Medicine, Yancheng Third People’s Hospital, The Affiliated Yancheng Hospital of Southeast University Medical College, Yancheng, Jiangsu China

**Keywords:** Bronchiectasis, Cardiovascular disease, Exacerbation, Clinical impact

## Abstract

**Background:**

Patients with bronchiectasis have a higher cardiovascular risk than their matched controls. However, the effect of cardiovascular (CV) disease on bronchiectasis remains unclear. Thus, we aimed to investigate the clinical impacts of cardiovascular disease on adult patients with bronchiectasis.

**Methods:**

The study cohort comprised 603 consecutive inpatients diagnosed with bronchiectasis in the Affiliated Yancheng Hospital of Southeast University Medical College (Jiangsu, China) from January 2014 to December 2017. Symptoms, bacterial cultures, blood biochemical indicator levels, and chest high-resolution computed tomography scans were assessed during their initial hospitalization for bronchiectasis. Three hundred and thirty five subjects finished 1 year follow-up after their hospital discharge.

**Results:**

Three hundred thirty five patients had at least one bronchiectasis exacerbation during the 1-year follow-up period. Patients with CV comorbidities were more likely to present with symptoms of wheezing (65.3%) and had a higher levels of brain natriuretic peptide (*P* < 0.001) and D-dimer (P < 0.001) than those without CV comorbidities. Independent risk factors associated with bronchiectasis exacerbations were the presence of comorbidities of cardiovascular diseases (odds ratio [OR] 2.503, 95% confidence interval [CI] 1.298–4.823; *P* = 0.006), the isolation of *Pseudomonas aeruginosa* (OR 2.076, 95% CI 1.100–3.919; *P* = 0.024), and extension to more than two lobes (OR 2.485, 95% CI 1.195–5.168; *P* = 0.015).

**Conclusion:**

The existence of cardiovascular disease was independently associated with increased bronchiectasis exacerbation.

## Background

Bronchiectasis is defined as the irreversible dilatation and thickening of bronchi, and it is diagnosed using high-resolution computed tomography (HRCT) as the recognized gold standard [1, 2]. Advances in CT technology have improved the detection and characterization of bronchiectasis and its complications [3]. Although the prevalence remains unknown in most areas, research has shown an increase in the incidence and prevalence of bronchiectasis [4]. Exacerbations that present with acute deterioration and worsening of local symptoms usually lead to hospitalization [1, 2, 5].

Comorbidities including cardiovascular (CV) disease, rheumatoid arthritis, and chronic kidney disease are commonly present in patients with bronchiectasis and significantly contribute to the disease burden and mortality [6]. Several studies have suggested a high prevalence of CV disease [7] and cardiac dysfunction [8] in bronchiectasis patients. Previous study has also shown increased arterial stiffness in patients with bronchiectasis, compared to their matched controls [9], and these patients have a higher risk of CV disease [10–12]. Interestingly, cardiovascular disease seemed to be linked to bronchiectasis, as it is to other diseases that feature increased systemic inflammation [13]. Previous research has reported a relationship between elevated cardiovascular risk and higher exacerbation frequency in chronic obstructive pulmonary disease (COPD) [14, 15], in addition, the calcification of coronary artery is raised in COPD patients and is related to a higher morbidity and mortality [16]. However, few studies have assessed the impact of CV disease on the exacerbation of bronchiectasis.

Research is warranted to obtain a deeper understanding of the relationship between CV disease and bronchiectasis and further reduce the risk of acute exacerbations in bronchiectasis. Therefore, this study aimed to evaluate the clinical characteristics of bronchiectasis patients with and without CV disease, and analyse the impacts of CV disease on bronchiectasis exacerbation.

## Methods

### Subjects

A retrospective cohort study was conducted on in-patients diagnosed with bronchiectasis in the Affiliated Yancheng Hospital of Southeast University Medical College (Jiangsu, China) from January 2014 to December 2017. Bronchiectasis was confirmed based on a radiological diagnosis established using HRCT and a clinical history consistency. Patients who had not undergone a chest HRCT scan examination or had indecipherable HRCT scan images were excluded. Patients with active malignancy, cystic fibrosis, significant immunodeficiency, or traction bronchiectasis resulting from pulmonary fibrosis/sarcoidosis were also excluded. Patients were also excluded if they had received long-term oral or inhaled antibiotic therapy. The study was approved by the Yancheng Third People’s Hospital ethics committee and was executed following the relevant guidelines and regulations.

### Diagnosis of bronchiectasis and CV disease

The diagnostic criteria for bronchiectasis are based on chest HRCT scans and clinical symptoms (coughing and expectoration or long durations of hemoptysis). During full inspiration, high-resolution scans from the apex to the base of the lungs were obtained at 1- milimeter collimation and 10- milimeter intervals. Bronchiectasis was diagnosed according to the following criteria: 1) a lack of bronchi taper 2) the internal diameter of bronchi dilation was greater than the next pulmonary artery or 3) within 1 cm of the costal or the adjacent mediastinal pleural surface, a visualization of the peripheral bronchi [17, 18]. The type of bronchiectasis was conformed morphologically. The bronchiectasis exacerbation was diagnosed based on deterioration in three or more of the following main symptoms for 48 h at least: cough, sputum volume or sputum consistency, purulent sputum, breathlessness or exercise tolerance, fatigue or malaise, hemoptysis, and the requirement of a change in the bronchiectasis treatment as determined by a clinician [19]. CV disease was a composite outcome of having a history of coronary heart disease (CHD) (acute coronary syndromes, chronic coronary artery disease), cerebrovascular events (including ischemic stroke, hemorrhagic stroke, or transient ischemic attack), peripheral artery disease or heart failure [20].

### Variables

We collected information regarding the following variables in this study: general and anthropometric information (i.e., age, gender, body mass index); smoking history; a history of having respiratory illness (e.g., nasosinusitis, tuberculosis [TB], pneumonia.) and clinical manifestation (e.g., symptom onset, chronic expectoration properties, the presence of wheezing); a history of comorbidities (e.g., coronary heart disease, hypertension, diabetes); chest HRCT scan (the number of bronchiectatic lobes); laboratory parameters (e.g., C-reactive protein, erythrocyte sedimentation rate, neutrophil percentage); and sputum microbiological examination (sputum specimens were eligible if they contained < 10 squamous epithelial cells and > 25 leukocytes per low-powered field). Exacerbations were recorded in bronchiectasis patients within 1 year after their hospital discharge, using face-to-face interviews during their outpatient service.

### Statistics

The statistical package SPSS version 22.0 was used for statistical analyses, and GraphPad Prism 5 Software was used for drawing graphs. All quantitative variables were listed as the mean ± standard deviation (SD) values, and the qualitative variables were listed as absolute numbers and percentages. The distribution of the variables was analysed using the Kolmogorov-Smirnov test. In the bivariate analysis, the t-test for independent variables was used to analyse the variables with normal distribution, and the Mann-Whitney U test was used in other cases. The chi-squared test was used to compare qualitative variables. Depending on whether the variables were distributed normally or non-normally, the correlation between variables was assessed by calculating Spearman or Pearson coefficient. In the case of elevated collinearity between two variables (Spearman correlation test > 0.6), the variable with greater clinical significance was included in the final regression eq. A logistic regression model was used to evaluate the related factors of bronchiectasis exacerbation. The variables that presented statistically significant differences (*p* < 0.05) in the bivariate analysis were included as the independent variables in the model. Thereafter, using the forward stepwise technique (the Wald test) to remove any variables with *p* > 0.1 from the final model. The odds ratio (OR) and 95% confidence interval for independent variables were calculated.

## Results

A total of 603 bronchiectasis patients (404 with only bronchiectasis and 199 bronchiectasis patients with CV disease) were included in the study. Among these 603 patients, 345 of the included patients completed a 1-year follow-up. One hundred forty-two patients had at least one bronchiectasis exacerbation within 1 year of hospital discharge.

The CV comorbidities and baseline characteristics of the patients are shown in Tables 1 and 2; the laboratory parameters are shown in Tables 3. There were several significant differences in the previous history, clinical symptoms, radiological signs, and blood biochemical indicator levels of the two groups. More patients (20.6%) in the group with co-existing CV disease tended to have a history of TB. Patients with CV comorbidities most commonly presented with symptoms of wheezing (65.3%) whereas those patients with no CV comorbidity more frequently presented with hemoptysis (44.6%). Further, patients with CV comorbidities had a higher level of brain natriuretic peptide and D-dimer, which are important indicators for assessing the severity of CV disease. Although there were significant differences in the levels of PO_2_ and PCO_2_ between the two groups, no significant clinical value was observed.

**Table 1:** Cardiovascular comorbidities in bronchiectasis patients

CV comorbidities	No. (%)
CHD	
acute coronary syndromes	81 (40.70%)
chronic coronary artery disease	23 (11.56%)
Cerebrovascular events	
ischemic stroke	37 (18.59%)
hemorrhagic stoke	8 (4.02%)
transient ischemic attack	58 (29.15%)
Peripheral artery disease	24 (12.06%)
Heart failure	29 (14.57%)

Total number of comorbidities add up to greater than the total number of patients because some patients developed more than one comorbidity

**Table 2:** Baseline and clinical characteristics of subjects with bronchiectasis, with and without cardiovascular disease

Parameter	Bronchiectasis (*n* = 404)	Bronchiectasis with cardiovascular disease (*n* = 199)	*P*-value
Age, years	61.97 ± 11.17	65.35 ± 9.75	0.107
Sex male: female n	191:213	80:119	0.1
BMI, kg/m^2^	23.81 ± 3.45	23.51 ± 3.22	0.591
Smoking	17.6%	16.6%	0.762
Previous pneumonia	38.9%	33.2%	0.173
Previous tuberculosis	12.4%	20.6%	**0.008**
Previous anaphylactic rhinitis	2.2%	1.5%	0.552
Presenting symptoms			
Cough	70.3%	73.4%	0.433
Wheezing	51.2%	65.3%	**0.001**
Hemoptysis	44.6%	34.7%	**0.02**
*P.aeruginosa* isolation	22.8%	30.2%	0.063
Type			
Cylindrical	28.2%	22.6%	0.142
Cystic	35.1%	40.7%	0.184
Mixed	36.6%	36.7%	0.99
Location			
Unilateral	25.0%	26.1%	
Bilateral	75.0%	73.9%	0.764
Extent			
Affected lobes n	3.21 ± 1.47	3.12 ± 1.43	0.465
Affected segments n	8.50 ± 5.15	8.71 ± 4.70	**0.011**

Data are presented as the mean ± SD or %, unless otherwise stated. *BMI* body mass index. Data presented in bold type are statistically significant

**Table 3:** Laboratory parameters of subjects with bronchiectasis with and without cardiovascular disease

	Bronchiectasis (*n* = 404)	Bronchiectasis with cardiovascular disease (*n* = 199)	*P*-value
WBC, ×10^9^ cells/L	8.40 ± 5.03	8.82 ± 4.11	0.551
Neutrophils, %	72.59 ± 12.05	75.48 ± 10.10	**0.003**
Haemoglobin, g/L	123.05 ± 19.74	125.56 ± 22.44	0.329
Platelet, ×10^9^ cells/L	196.89 ± 83.96	195.24 ± 88.47	0.480
CRP, IU/mL	28.53 ± 44.69	29.89 ± 38.82	0.168
ESR, mm/h	41.07 ± 29.18	36.03 ± 30.61	0.168
Albumin, mg/dL	37.90 ± 5.09	36.86 ± 4.28	0.095
K^+^, mmol/L	3.98 ± 0.47	4.05 ± 0.61	**0.009**
Na^+^, mmol/L	140.18 ± 4.51	139.52 ± 4.30	0.468
Cl^−^, mmol/L	99.41 ± 5.23	96.09 ± 7.10	**0.001**
Ca^+^, mmol/L	2.22 ± 0.36	2.20 ± 0.14	0.697
BUN, mol/L	5.55 ± 3.35	6.04 ± 3.36	0.069
Blood glucose, mmol/L	6.23 ± 2.47	6.56 ± 2.58	0.062
BNP, pg/L	1365.22 ± 2996.11	3602.01 ± 6297.81	**< 0.001**
Cholesterol, mol/L	4.09 ± 0.97	4.16 ± 1.84	0.171
Triglyceride, mol/L	1.16 ± 0.67	1.13 ± 0.60	0.772
D-dimer, mg/L	0.69 ± 1.105	1.00 ± 1.676	**< 0.001**
PO_2_, kPa	10.11 ± 3.20	10.26 ± 3.50	**0.018**
PCO_2_, kPa	6.59 ± 1.69	7.34 ± 1.83	**0.02**
SO_2_, %	92.95 ± 7.55	92.19 ± 7.15	0.271

Data are presented as the mean ± SD or %, unless otherwise stated. *WBC* white blood count, *CRP* C-reactive protein, *ESR* erythrocyte sedimentation rate, *BUN* serum urea nitrogen, *BNP* brain natriuretic peptide, *PO2* oxygen tension, *PCO2* carbon dioxide tension, *SO2* oxygen saturation. The data presented in bold type are statistically significant

**Fig. 1:**
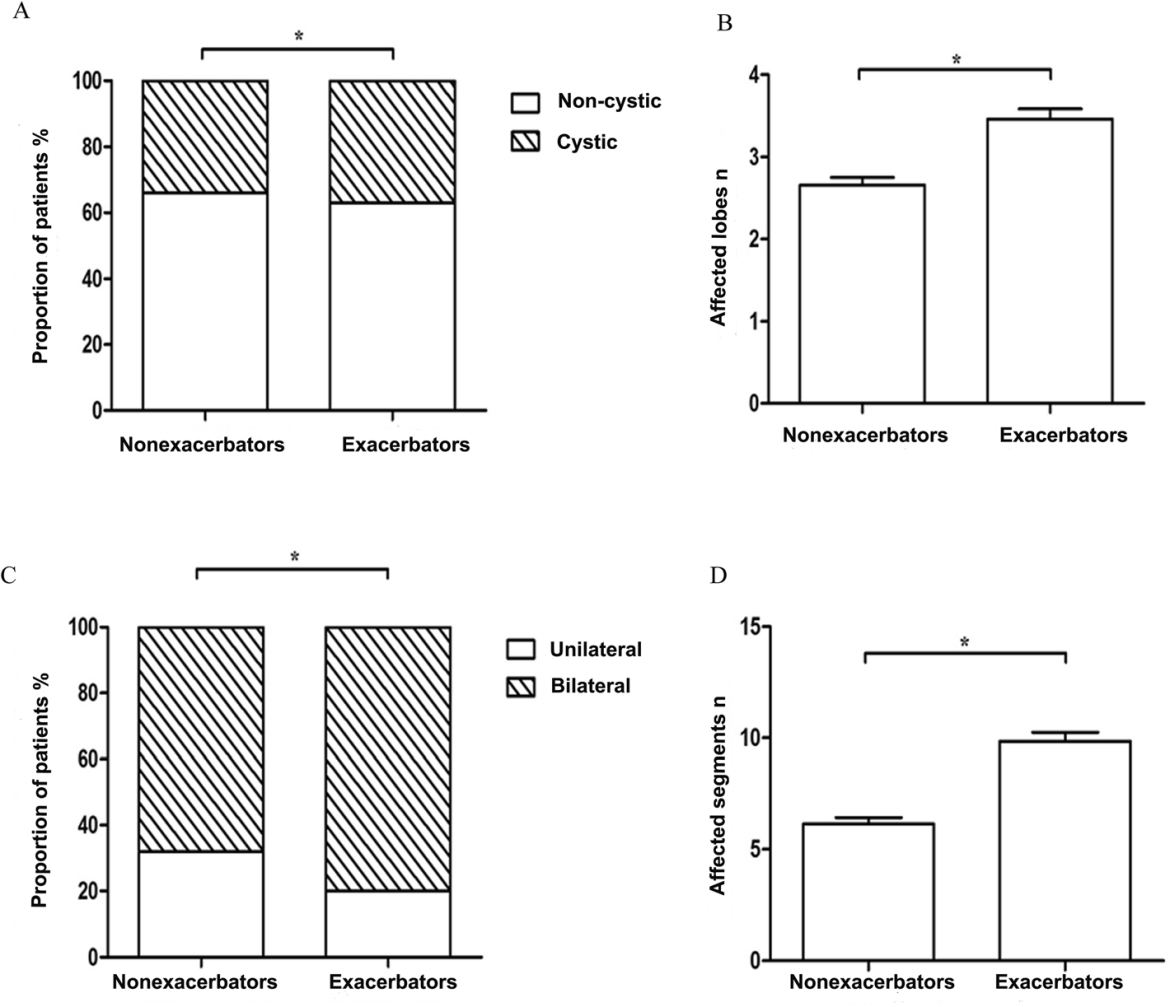
**a** Proportion with cystic bronchiectasis; **b** number of affected lobules; **c** proportion with bilateral location; and (**d**) number of affected segments of patients who experienced exacerbation (at least once) and that of patients who did not experience an exacerbation. *: *P* < 0.05

**Fig. 2:**
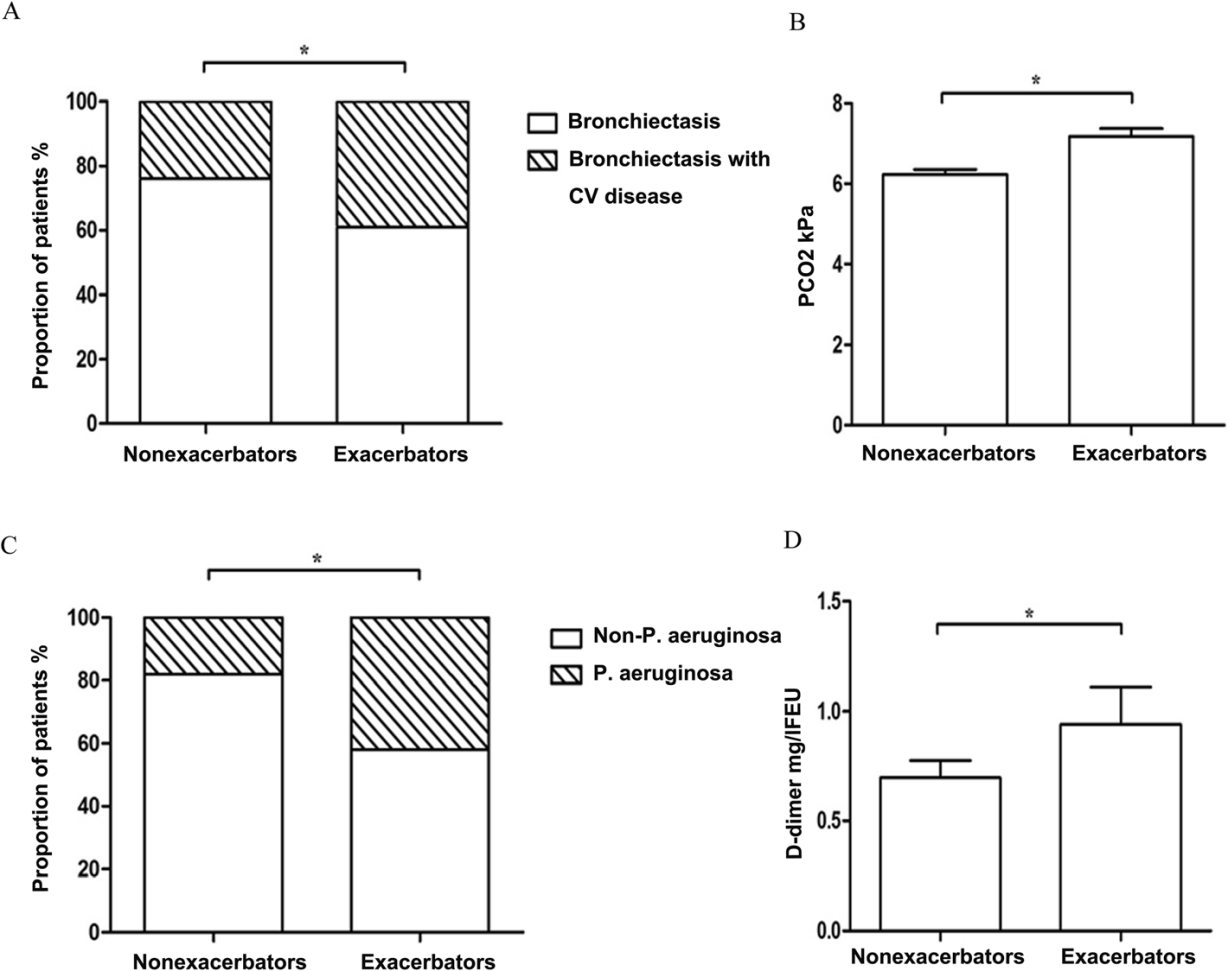
**a** Proportion with cardiovascular disease; **b** level of PCO_2_; **c** proportion with *P. aeruginosa* isolation; and (**d**) level of D-dimer of patients who experienced at least one exacerbation and that of patients who did not experience an exacerbation. *: *P* < 0.05

Figures 1 and 2 show the differential characteristics of patients with at least one exacerbation and patients with no exacerbation. Patients with one or more exacerbations had worse HRCT scan images that showed more cystic bronchiectasis and involved a wider region of affected lobes or segments, a higher prevalence of CV disease comorbidities, more positive cultures of *P.aeruginosa* isolation, and higher levels of PCO_2_ and D-dimer than patients with no exacerbation..

Table 4 summarizes the ORs and 95% confidence intervals of variables related to bronchiectasis exacerbations in all patients. The variables with OR = 1 involve no risk of bronchiectasis exacerbation. The presence of CV disease comorbidities (*P* = 0.006), *P.aeruginosa* isolation (*P* = 0.024), and extension to more than two lobes (*P* = 0.015) were independent risk factors for bronchiectasis exacerbation in these patients.

**Table 4:** Factors associated with bronchiectasis exacerbation in all subjects according to the logistic regression analysis

	OR (95% CI)	*P*-value
Co-existing CV diseases	2.503 (1.298–4.823)	**0.006**
*P. aeruginosa* isolation	2.076 (1.100–3.919)	**0.024**
Cystic	1.369 (0.695–2.697)	0.364
Bilateral	0.929 (0.394–2.188)	0.866
Extent > 2 lobes	2.485 (1.195–5.168)	**0.015**
Hypercapnia	1.589 (0.881–2.864)	0.124
Higher D-dimer level	1.232 (0.630–2.410)	0.542

Factors associated with bronchiectasis exacerbation in all subjects according to the logistic regression analysis. *CV* diseases: cardiovascular diseases; *P. aeruginosa Pseudomonas aeruginosa*

## Discussion

The key findings of our study suggest that in non-cystic fibrosis bronchiectasis patients, the presence of cardiovascular diseases, the isolation of *P.aeruginosa* from sputum samples, and extension to more than two lobes were associated with an increased risk of bronchiectasis exacerbation.

This is a cross-sectional and observational study on the prevalence of CV disease with a large number of bronchiectasis patients from China. Several studies have also reported a high prevalence of CV disease in patients with bronchiectasis that varies according to the population that is analysed [7–9]. A historical cohort analysis has demonstrated a causal link between the two diseases, suggesting a higher risk of CV disease in patients with bronchiectasis [10–12]. Moreover, excess CV risk is associated with greater bronchiectasis severity [11], frequency deterioration, impaired lung function [12]. Our results confirmed a high prevalence of CV disease in bronchiectasis patients, which is consistent with published research.

The possible mechanisms for the increased CV disease prevalence need further exploration. Bronchiectasis is characterized by chronic inflammation and dysfunction of clear airway secretions, leading to recurrent infection. Previous studies have reported that bronchiectasis patients have increased systemic inflammation [21–23], such as increased vascular adhesion molecules [24, 25], which plays a crucial role in the development of atherosclerosis [26] and is associated with vulnerable atherosclerotic plaque and subsequent thromboembolic events [27, 28]. Moreover, the higher prevalence of acute infections in bronchiectasis patients may also be associated with a transient increase in the risk of vascular events [29]. In addition, Gale and colleagues have shown increased arterial stiffness in bronchiectasis, which is a well-acknowledged risk factor for vascular disease [9].

We next explored the clinical characteristics of bronchiectasis subjects with and without cardiovascular disease. The group of patients who had bronchiectasis with CV disease were more likely to have a history of previous tuberculosis, which is consistent with recent epidemiological work that the risk of CVD in persons who develop tuberculosis is higher than that in persons without a history of tuberculosis [30–33]. Together, these data indicate that tuberculosis may play a part in the CVD pathogenesis, and further research is necessary to investigate the potential connection between tuberculosis, bronchiectasis and CVD. In the current study, we also found that patients with CV comorbidities had a higher level of brain natriuretic peptide and D-dimer. Brain natriuretic peptide (BNP) is a marker of myocardial and circulatory stress that can predict future cardiac events and death in asymptomatic populations [34]. The natriuretic peptides have an essential role in vascular function and remodeling by increasing nitric oxide effects [35], inhibiting lipid insulation in the vascular wall [36], and increasing parasympathetic tension [37]. Plasma D-dimer, a fibrin degradation product, is another important predictor of stroke [38, 39], CHD [38], VTE [40, 41], and CVD [42]. However, between D-dimer and cardiovascular risk factors, their pathophysiology has not been fully comprehended. Neil et al. found that the association of D-dimer with cardiovascular disease did not depend on elevated inflammatory biomarkers, demonstrating that D-dimer may shed light on different pathophysiologies of cardiovascular disease by type and race [39]. Future studies are needed to address the role played by brain natriuretic peptide and D-dimer in bronchiectasis.

The exacerbation of bronchiectasis is a crucial target for therapy given that it is a major determinant of the cost of healthcare [1]. Further, more severe and frequent exacerbations are associated with mortality [43]. Exacerbations are associated with increased airways and systemic inflammation [25] and progressive lung damage [43, 44], the exacerbations can be explained according to the vicious cycle of chronic bronchial infection, inflammation, impaired mucociliary clearance and structural lung damage [1]. In this study, the extent of bronchiectasis based on radiography and the isolation of *P.aeruginosa* from the sputum samples were associated with bronchiectasis exacerbation. The degree of bronchiectasis, quantified according to the number of affected lobes, was included in the analysis. This is consistent with the result of a previous study wherein pulmonary extension could be used to evaluate the severity of bronchiectasis [45]. *P.aeruginosa* was an independent factor associated with bronchiectasis exacerbation in the present study, which is supported by previous researches [46, 47]. In our study, compared with bronchiectasis alone, patients with CV disease were 2.503 times more likely to experience exacerbation, independent of other variables. The underlying reason for increased exacerbation in bronchiectasis with CV disease remains unclear, it could be because they share similar risk factors, such as systemic inflammation or acute infection. Further studies are warranted to demonstrate the biological mechanism between CV disease and the exacerbation of bronchiectasis.

There are certain limitations of this study. The first limitation was the possibility of selection bias due to the retrospective study design. Thus, we conducted a cross-sectional and observational study that may minimize the risk of selection bias. Second, pulmonary function test results are shown to be associated with poor prognosis in bronchiectasis patients [48]; however, this study does not include pulmonary function tests. A previous study has shown that serial CT changes were correlated with the pulmonary function trends [44]; hence, we used CT findings to evaluate the severity of bronchiectasis. Third, we did not consider the treatment status in the analysis for patient compliance; this may have affected the results. Finally, the generalizability of our findings may be limited because of the single-centre design; therefore, our findings require additional validation, including data from other countries and institutions.

## Conclusion

In summary, our results suggest that the existence of cardiovascular disease was independently associated with increased bronchiectasis exacerbation. Awareness and the mitigation of existing cardiovascular disease may have the potential to reduce exacerbation and require further study to improve clinical outcomes in bronchiectasis.

## Data Availability

The datasets used and/or analysed during the present study are available from the corresponding author on reasonable request.

## Abbreviations

CV: Cardiovascular
OR: Odd’s ratio
CI: Confidence interval
HRCT: High-resolution computed tomography
TB: Tuberculosis
